# The Transcription Factor *Elf3* Is Essential for a Successful Mesenchymal to Epithelial Transition

**DOI:** 10.3390/cells8080858

**Published:** 2019-08-09

**Authors:** Burcu Sengez, Ilkin Aygün, Huma Shehwana, Neslihan Toyran, Sanem Tercan Avci, Ozlen Konu, Marc P. Stemmler, Hani Alotaibi

**Affiliations:** 1 Izmir Biomedicine and Genome Center, 35340 Izmir, Turkey; 2 Izmir International Biomedicine and Genome Institute, Dokuz Eylül University, 35340 Izmir, Turkey; 3 Department of Biological Sciences, National University of Medical Sciences, Rawalpindi 46000, Pakistan; 4 Vocational School of Health Services, Dokuz Eylül University, 35340 Izmir, Turkey; 5 Department of Molecular Biology and Genetics, Bilkent University, 06800 Ankara, Turkey; 6 Department of Experimental Medicine 1, Nikolaus-Fiebiger Center for Molecular Medicine, Friedrich-Alexander University Erlangen-Nürnberg, D-91054 Erlangen, Germany

**Keywords:** mesenchymal to epithelial transition, *Elf3*, *Grhl3*, Cadherins

## Abstract

The epithelial to mesenchymal transition (EMT) and the mesenchymal to epithelial transition (MET) are two critical biological processes that are involved in both physiological events such as embryogenesis and development and also pathological events such as tumorigenesis. They present with dramatic changes in cellular morphology and gene expression exhibiting acute changes in E-cadherin expression. Despite the comprehensive understanding of EMT, the regulation of MET is far from being understood. To find novel regulators of MET, we hypothesized that such factors would correlate with *Cdh1* expression. Bioinformatics examination of several expression profiles suggested *Elf3* as a strong candidate. Depletion of *Elf3* at the onset of MET severely impaired the progression to the epithelial state. This MET defect was explained, in part, by the absence of E-cadherin at the plasma membrane. Moreover, during MET, ELF3 interacts with the *Grhl3* promoter and activates its expression. Our findings present novel insights into the regulation of MET and reveal ELF3 as an indispensable guardian of the epithelial state. A better understanding of MET will, eventually, lead to better management of metastatic cancers.

## 1. Introduction

The epithelial to mesenchymal transition (EMT) and the mesenchymal to epithelial transition (MET) has been known to scientists for decades. They are crucial for a variety of biological processes such as embryogenesis, development, and wound healing [1,2,3], and their aberrant regulation has deleterious outcomes [1,4,5,6]. For example, tumor cells of epithelial origin can benefit from an EMT event and lose their adherent and polarized morphology and become motile and unpolarized [1,7]. These cells can now relocate and colonize sites in distant organs. To accomplish a metastatic status, relocated tumor cells need to undergo a MET event, after which, they would regain the polarized epithelial-like state, proliferate, and form secondary tumors [1,6,8]. 

Alongside the global changes in gene expression and cytoskeletal rearrangements, switching the expression of the adhesion molecules E-cadherin and N-cadherin is a hallmark of EMT and MET events [3,6,8,9,10]. The downregulation of E-cadherin is crucial for a successful EMT [10,11], which is accomplished by a group of transcription factors including but not limited to, *Snai1*, *Snai2*, *Zeb1*, and *Zeb2* [3,12], collectively dubbed ‘The EMT Inducers’. These transcription factors bind to several E-boxes in the promoter of *Cdh1* and cause the repression of E-cadherin expression [12,13,14,15]. The initiation of MET has been mostly explained as a result of the reduction of the levels of the EMT inducers [15,16]; however, MET is not merely the reverse process of EMT and is a fundamental program as well [2,17]. It involves a series of dynamic and tightly regulated events by either transcriptional and posttranscriptional mechanisms, in the center of which is the initiation and maintenance of *Cdh1* expression and the presence of E-cadherin at the plasma membrane [18], which are essential steps for proper entry to MET [10]. MET is primarily described as a loss of the expression of transcription factors such as *Snai1/2* and *Zeb1/2* and gaining epithelial-like features via the initiation of *Cdh1* and *Epcam* expression. Previous studies have shown that E-cadherin internalization and trafficking to and from the plasma membrane is crucial for maintaining epithelial dynamics [18,19] and controlling protein stability at the cell surface and cellular levels of the protein [15].

The hallmarks of transitions between epithelial and mesenchymal states include changes in the cadherin repertoire [20,21,22]. The *Cdh1* promoter alone, including all known E-box elements, is insufficient to confer a strict cell-type specificity [23]. In contrast, intron 2 carries sufficient information for proper E-cadherin expression [24]. Although the second intron has emerged as an essential regulator of *Cdh1* expression [23,24,25], very little is known about the molecular determinants controlling the transcriptional activity of the locus. Besides *Klf4* [26,27], Grainy head-like 2 (*Grhl2*), a mammalian homolog of Drosophila grainy head, was shown to control epithelial differentiation in several tissues [28,29,30] and during uretic bud formation by regulating E-cadherin via binding to an element in intron 2 [31].

Moreover, the GRHL3 transcription factor plays a fundamental role during the initiation of MET [25]. It activates the expression of *Hnf4α*, and together they cooperate to activate the expression of *Cdh1* through binding to enhancers in the second intron. Despite the abundance of publications on the regulation of *Cdh1* expression, there still exists limited knowledge on the transcription factors involved in the regulation of *Cdh1* expression. It is then plausible to assume that regulators of *Cdh1* transcription are de facto regulators of MET; thus, a better understanding of how *Cdh1* is transcriptionally regulated would shed light on the regulation of MET. 

To gain insights into this, we searched for novel regulators of MET and hypothesized that regulators of MET could be regulators of *Cdh1* transcription or at least have a strong correlation with its expression. Using an in silico approach, we first generated an epithelial-specific set of transcription factors with high correlation to *Cdh1* expression. Moreover, we used the normal murine mammary gland epithelial cells (NMuMG) that undergo a reversible EMT by transforming growth factor β (TGFβ) treatment and identified *Elf3* as a compelling regulator of MET and E-cadherin localization to the plasma membrane. Moreover, we provide evidence showing that ELF3 is a regulator of the transcription factor *Grhl3*.

## 2. Materials and Methods

### 2.1. Microarray Data Analysis and Correlation Study

Raw data (CEL files) of four gene expression datasets based on the GPL6246 platform (GSE130548, GSE77551, GSE55711, and GSE55072) were downloaded from the Gene Expression Omnibus database. Samples with distinct epithelial and mesenchymal states were selected for analysis. Datasets and samples included in the analysis are shown in Table S1. Data were RMA normalized using Bioconductor package oligo [32]. Normalized data were merged and transformed into z-scores using scale function in R [33]. The z-score transformation was performed both on the sample and on gene level [34]. Merged z-scores profile was used to evaluate spearman correlation between *Cdh1* and all other probe sets present in the expression profile. Differential expression analysis between epithelial and mesenchymal samples was performed using the Limma package [35] to retrieve log fold changes and p-values. Gene to probe set annotation was performed using the annotate package [33] and mogene10sttranscriptcluster.db annotation library [36]. List of mouse transcription factors was retrieved from Animal TFDB3.0 [37]. *Cdh1* correlation and differential expression profile of transcription factors were extracted from the performed analysis. The Ggplot2 package was used to draw the plots [38].

### 2.2. Cell Culture and Induction of EMT and MET

NMuMG (ATCC^®^ CRL-1636) cells were cultured in DMEM (11965092, Gibco, Waltham, MA, USA) supplemented with 10% fetal bovine serum (FBS, 10500064, Gibco), penicillin-streptomycin (10,000 U/mL, 15140122, Gibco), 1× MEM NEAA (1140035, Gibco), and 10 µg/mL insulin (19278-5 mL, Sigma, Munich, Germany). NMuMG cells were grown at 37 °C in a humid environment with 10% CO_2_. MEFs were isolated from C57BL/6J E13.5 embryos and cultured in DMEM supplemented with 10% fetal bovine serum, penicillin-streptomycin (10,000 U/mL), and 1× MEM NEAA. Cells were grown at 37 °C in a humid environment with 5% CO_2_. Keratinocytes were isolated from the back skin of P0 newborn mice as described previously [39], and cultured in Epidermal Keratinocyte Medium (CnT-07, CellnTec, Bern, Switzerland) containing penicillin-streptomycin (10,000 U/mL) and supplements A, B, and C (CellnTec, Bern, Switzerland). Cells were grown at 37 °C in a humid environment with 10% CO_2_. Manipulations using animals were approved by the Animal Care and Use Committee of Izmir Biomedicine and Genome Center (iBG, Izmir, Turkey). For EMT induction, cells were maintained in complete medium supplemented with five ng/mL TGFβ3 (100-36E, PeproTech, London, UK) for 72 h (labeled as TGFβ3). The induction of MET was initiated by TGFβ3 withdrawal and washing the plates twice with PBS and incubation for an additional 72 h in fresh medium (labeled PT as in Post-treatment). 

### 2.3. siRNA Knockdown and mRNA Expression Analysis

siRNAs against Elf3 were purchased from Qiagen for siElf3_2 and siEhf_1-4, and from Dharmacon for siElf3_10 and siElf3_12; sequences are available in Table S2. Cells were transfected with 50 nM siRNA using the Lipofectamine RNAi Max (13778150, Invitrogen, Waltham, MA USA) and collected 72 h later. Total RNA was isolated using the Nucleospin RNA II kit (740955.50, Macherey-Nagel, Düren, Germany) as recommended by the manufacturer. Total RNA (1 µg) was converted to cDNA using the Maxima First Strand cDNA Synthesis Kit (K1642, Thermo Scientific, Waltham, MA USA). cDNA was then diluted 1:20 and used for qPCR with the TaqMan™ Universal Master Mix II (4440040, Applied Biosystems, Waltham, MA USA) and Universal Probe Library probes (Roche, Basel, Switzerland). All qPCR data were normalized to the *Gapdh* gene. Primers and probes used are available in Table S3. Quantification was performed using the ΔΔCt method.

### 2.4. Luciferase Reporter Assays

Cells in 48-well plates were transfected with plasmid DNAs using Fugene HD transfection reagent (E2311, Promega, Madison, WI, USA). Transfection was carried out with 100 ng DNA containing five ng pRL-TK (Promega) to normalize for transfection efficiency. We normalized to equal molarity of the plasmid to use equivalent copies of the reporter plasmid. The total DNA content was complemented with promoter-less plasmid DNA. 20 ng of *Elf3* expression plasmid or empty vector (Mock) were transfected. Luciferase reporter activity was measured using Dual-Glo Luciferase Assay (E2940, Promega, Madison, WI, USA) in a Centro XS³ LB 960 luminometer (Berthold Technologies, Bad Wildbad, Germany). Firefly luciferase reporter values were normalized to those of the Renilla luciferase control. Fold induction was calculated relative to the values of the empty vector.

### 2.5. Expression and Reporter Vectors

The *Elf3* expression vector was prepared by amplifying the coding sequence of *Elf3* by PCR, adding restriction sites for *Bam*HI (5’) and *Eco*RI (3’). The PCR product was inserted into a modified version of pcDNA4/TO/Myc-His A (Invitrogen, Waltham, MA USA) containing a sequence coding for a 3× FLAG epitope between *Hin*dIII and *Bam*HI sites. The full sequence of the plasmid is available upon request.

The *Grhl3* promoter (1 kb; between −1 and −1000 bp relative to the transcription start site) was amplified by PCR in 25 µL reaction volume containing 1× PCR buffer, 1.5 mM MgCl_2_, 200 µM dNTP, 10 pmol of each primer (Table S4), 5% DMSO, 1 U of *Taq* DNA polymerase (2900242, 5 PRIME), and 50 ng of C57BL/6J mouse genomic DNA. Thermal cycler conditions were an initial denaturation step at 95 °C for 5 min, 30 cycles of 95 °C for 30 s, 60 °C for 30 s, and 72 °C for 30 s, followed by a final extension at 72 °C for 10 min. The amplified PCR products were subsequently inserted upstream of the luciferase reporter in pGL4.10 (Promega, Madison, WI, USA) using *Xho*I and *Hin*dIII restriction sites to produce the reporter plasmid named pGrhl3. The shorter versions of pGrhl3 were prepared by removing sequences flanked by *Xho*I/*Bsa*I for pGrhl3-XB, *Xho*I/*Psp*1406I for pGrhl3-XP and *Psp*1406I/*Hin*dIII for pGrhl3-PH.

### 2.6. Chromatin Immunoprecipitation (ChIP)

ChIP was performed as reported previously [40] with the following minor modifications. NMuMG cells were grown and treated in 10 cm plates. Following 10 min cross-linking and lysis, cell lysates were sonicated in the S220 focused-ultrasonicator (Covaris, Woburn, MA, USA) for 30 min (60 s on/60 s off; Peak Power: 140 W; Duty factor: 15; Cycles/burst: 200; water temperature: 4 °C). Immunoprecipitation was performed at 4 °C for 16 h using an antibody against ELF3/ESE-1 (NBP1-30873, Novus Biologicals, Centennial, CO, USA) and a rabbit control IgG (2729S, Cell Signaling, Danvers, MA, USA). Quantitative real-time PCR was performed using the TaqMan Universal master mix II and Universal Probe Library probes (Roche, Basel, Switzerland) and amplification was carried out in the ABI PRISM 7500 Fast qPCR System (Thermo Scientific, Waltham, MA USA) with primers and probes depicted in Table S5 at 95 °C for 15 min, 50 cycles of 95 °C for 15 s and 60 °C for 1 min. Data were normalized to the input and presented as percentage of input.

### 2.7. Immunofluorescence Labeling and Confocal Microscopy

Cells were washed with PBS and fixed with 4% formaldehyde (F8775, Sigma) for 10 min at room temperature, washed twice with PBS, and permeabilized with 0.25% Triton X-100 (T8787, Sigma) for 5 min. After washing twice with PBS, cells were incubated with primary antibodies in PBS with 5% BSA (1:200) for one hour at room temperature. Afterward, Alexa594-conjugated secondary antibody was applied for an additional one hour after washing in PBS. Nuclei were visualized with DAPI (1:1000, Invitrogen) and mounted with Prolong Diamond Antifade Mountant solution (P36962, Thermo Fisher Scientific, Waltham, MA USA). Confocal microscopy was carried out with an LSM 880 microscope equipped with ZEN software (Zeiss, Oberkochen, Germany). The antibodies used were anti-E-cadherin (610181, BD Bioscience, San Jose, CA, USA), Vimentin (11-254-C100, Exbio, Vestec, Czech Republic), Alexa Fluor™ 488 Phalloidin (A12379, Invitrogen), DAPI (Invitrogen), and Alexa Fluor 594 goat anti-mouse IgG (A-11005, Invitrogen, Waltham, MA USA)

### 2.8. SA-β-GAL Assay

NMuMG cells recovering from TGFβ3 were transfected with siCntrl or siElf3_10 as described before. Seventy-two hours later, the cells were washed with PBS, and SA-β-Gal activity was measured by a Senescence Detection Kit (K320, BioVision, Milpitas, CA, USA) according to the manufacturer’s instruction. As a positive control, Huh7 cells were used. Induction of senescence was performed with 100 nM Doxorubicin (Dox; A4361, Applichem, Darmstadt, Germany). Huh7 cells were grown in appropriate media containing Dox for three days and an additional six days after Dox withdrawal. The presence of senescent cells was detected using an Olympus CKX41 microscope equipped with a DP25 camera.

### 2.9. RT-PCR

Total RNA was isolated using the Nucleospin RNA II kit (Macherey-Nagel, Düren, Germany) as recommended by the manufacturer. Total RNA (1 µg) was converted to cDNA using the Maxima First Strand cDNA Synthesis Kit (Thermo Scientific, Waltham, MA USA). cDNA was then diluted 1:20 and 2 µL was used for PCR amplification of target genes in a 50 µl reaction containing 0.2 µM of forward and reverse primers (Table S6), 200 µM of dNTPs, 1 × Standard *Taq* (Mg-free) Reaction buffer, 1.5 mM MgCl_2_, and 1.25 units of *Taq* DNA polymerase (M0320S, New England Biolabs, Ipswich, MA, USA). PCR conditions were 95 °C for 5 min; 35 cycles of 95 °C for 30 s, 60 °C for 30 s and 72 °C for 1 min; 72 °C for 5 min. Amplified DNA was visualized by electrophoresis on a 2% agarose gel.

### 2.10. Fluorescence-Activated Cell Sorting (FACS)

TGFβ3 treated cells were prepared as described before. Cells (10 × 10^6^) were harvested, washed in FACS buffer (1 mM EDTA, 25mM HEPES and 1% FBS in PBS), and then stained with E-cadherin antibody (sc-8426, Santa Cruz Biotechnology, Dallas, TX, USA) and goat anti-mouse IgG (H + L) cross-adsorbed secondary antibody, Alexa Fluor 488 (A-11001, Thermo Fisher Scientific, Waltham, MA USA) for 30 min at 4 °C. Cells were washed with FACS buffer and stained with DAPI (final concentration: 0.1 µg/µL). Stained cells subsequently collected by BD FACSAria™ III and single E-cadherin-positive and E-cadherin-negative cells were collected in individual wells of a 96-well plate and allowed to grow into colonies.

### 2.11. Western Blot

NMuMG cells recovering from TGFβ3 were transfected with either siCntrl or siElf3_10 as described before. Seventy-two hours later, the cells were harvested and lysed in RIPA buffer. Protein concentration was determined by TaKaRa BCA Protein Assay Kit (T9300A, Takara, Kusatsu, Japan Protein samples were then diluted with Laemmli Sample buffer. After electrophoresis, proteins were transferred to a nitrocellulose membrane, blocked for 1 h in blotto (Tris-buffered saline containing 0.5% Tween 20 and 5% nonfat milk powder), and incubated with primary antibodies at 1:1000 dilution (Anti-E-cad: 610181, BD Biosciences and anti-ELF3: NBP1-30873, Novus Biologicals) overnight at 4 °C with shaking. Anti-GAPDH (sc-47724, Santa Cruz Biotechnology, Dallas, TX, USA) was used as a loading control. Proteins were detected with IRDye^®^ 800CW Goat anti-Mouse IgG and IRDye^®^ 680RD Goat anti-Mouse IgG (H + L) antibodies (LI-COR Biosciences, Lincoln, NE, USA) visualized on an Odyssey^®^ CLx Imaging System (LI-COR Biosciences, Lincoln, NE, USA).

### 2.12. Wound Healing Assay

For wound healing assay experiments, NMuMG cells recovering from TGFβ3 were transfected with either siCntrl or siElf3_10 as described before. Six hours later, cells were scratched with a sterile pipette tip and cultured for an additional 48 h. Microscopic evaluations in 24-h intervals were carried out using an Olympus CKX41 microscope equipped with a DP25 camera. Scratch width was determined by measuring the distance in pixels between the edges of the scratch in ImageJ. Several measurements were taken from each slide, and the average was calculated.

### 2.13. Statistical Analysis

Statistical significance was determined by performing the Student’s *t*-test using the normalized values of each test sample compared to the normalized value of the control, using a 95% confidence interval; *p*-values less than 0.05 were considered significant. For Figure S2A One Way ANOVA test was performed. Data are presented as the mean of at least three independent experiments, done in triplicates. Luciferase reporter assays and ChIP experiments were carried out at least three times, qPCR was performed in triplicates. Error bars represent standard error of the mean.

## 3. Results

### 3.1. The Transcription Factors Elf3 and Grhl3 Are Highly Correlated with the Epithelial State

Previously, we have reported that the transcription factor *Grhl3* is essential for the progression of MET and we showed that it is a crucial regulator of the cell adhesion molecule E-cadherin as well as the transcription factor *Hnf4α* [25]. In this study, we wanted to reveal other transcription factors essential for the progression of MET. We reasoned that the expression of such transcription factor(s) would correlate with that of *Cdh1*. To this end, we analyzed the expression profiles from several experimental microarray datasets originating from different and unrelated studies but contained distinct epithelial and mesenchymal samples. We had to apply some criteria to be unbiased in our selection, as the NCBI GEO database includes hundreds of examples. One of the data sets used here is an expression profile of primary keratinocytes and E13.5 MEFs previously produced in our lab (GSE130548 prepared with the Affymetrix MoGene-1_0-st chip (GEO platform GPL6246)). Since we wanted to compare epithelial and mesenchymal samples across different studies, using samples coming from the same platform would make the analyses relatively comparable. Second, the selected study should contain both epithelial and mesenchymal samples. The distinction between epithelial and mesenchymal phenotypes within the same study was made based on the expression of *Cdh1*, *Epcam*, *Fn1*, and *Vim* genes. In addition to our dataset (six samples), we were able to identify three datasets (GSE77551, GSE55711, and GSE55072) with 14 samples, ending up with a total of 20 samples.

**Figure 1:**
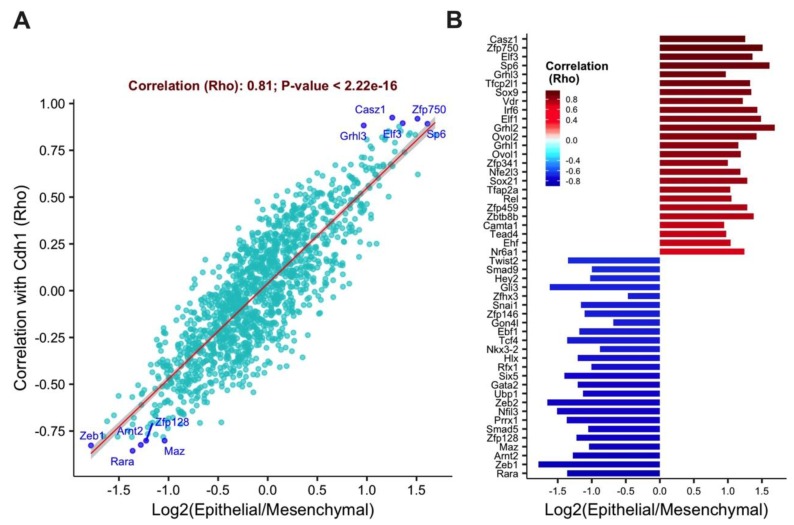
The transcription factors *Elf3* and *Grhl3* are highly correlated with the epithelial state. (**A**) A scatter plot that shows the correlation of 1469 transcription factors with the epithelial phenotype. Microarray-based screen for epithelial-specific transcription factors coregulated with E-cadherin. One-thousand-four-hundred-and-sixty-nine transcription factors were analyzed for differential expression in epithelial compared to mesenchymal samples, and Spearman correlation was calculated between each transcription factor and E-cadherin across all data sets. The top 5 correlated genes with either the epithelial or the mesenchymal phenotypes are labeled. (**B**) The ranking plot is displaying the top 25 correlated transcription factors in the epithelial and in the mesenchymal states. The x-axis represents the differential expression of each transcription factor in epithelial versus mesenchymal samples.

Correlation analysis of Z-score derived from 4 datasets revealed that the transcription factors *Casz1*, *Zfp750*, *Elf3*, *Sp6*, and *Grhl3* as highly correlated with *Cdh1* gene and highly expressed in the epithelial phenotype (Figure 1A), while transcription factors such as *Zeb1*, *Zeb2*, *Snai1*, and *Twist2* were found overexpressed in the mesenchymal phenotype. Top positively and negatively correlated genes are shown in Figure 1B. Genes highly correlated with *Cdh1* were indeed associated with upregulation in epithelial cell type as indicated by positive fold change, on the other hand, negatively correlated genes elevated in mesenchymal phenotype elicited by negative log fold change.

### 3.2. Elf3 Is Essential for MET

To establish the relevance of the highly correlated transcription factors to MET, we utilized NMuMG cells (an established murine model for the study of EMT and MET). These cells will gain a mesenchymal phenotype once treated with TGFβ for three days, and after TGFβ withdrawal, the cells will revert to the epithelial phenotype in three days (MET). We first analyzed the expression levels of the top correlated genes in NMuMG cells by RT-PCR (Figure S1A). While *Casz1* and *Zfp750* were barely detectable in NMuMG cells (only detectable when 20 times more cDNA was used), *Elf3* had comparably higher RNA levels, and thus was selected for further analyses. We used siRNA mediated silencing of candidate genes at the onset of MET in NMuMG cells and followed the progression of MET both morphologically as well as by expression levels of epithelial and mesenchymal defining genes.

**Figure 2:**
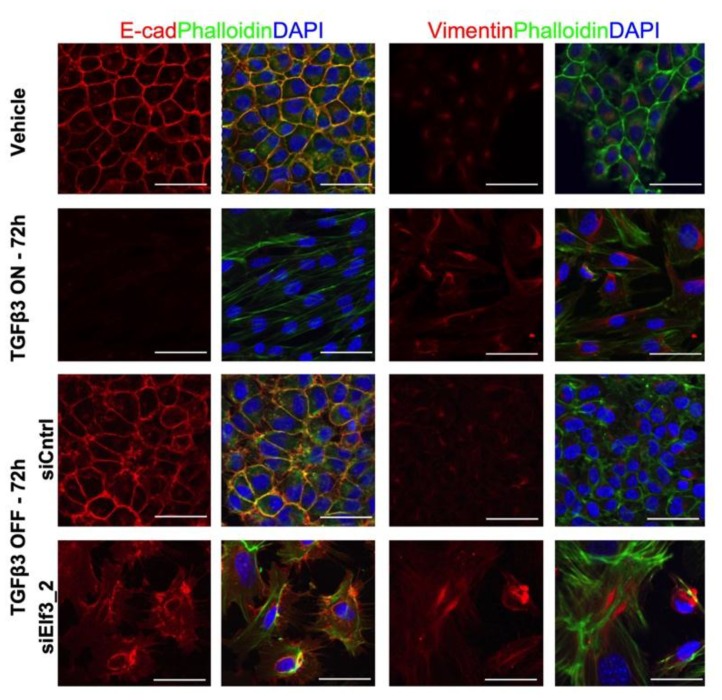
The siRNA-mediated silencing of *Elf3* result in an impaired MET. Confocal images showing the immunofluorescence staining of NMuMG cells visualizing changes in expression and intracellular distribution of E-cadherin, Vimentin, and Actin during TGFβ3 treatment, withdrawal, and also during the silencing of *Elf3*. Actin distribution (Phalloidin, green) and detection of E-cadherin (red) and Vimentin (red) is shown. Nuclei are labeled with DAPI. Scale bar, 50 μm.

We first validated MET in NMuMG cells by single-cell sorting of cells treated with TGFβ for three days, as well as seeding TGFβ treated cells at a very low density (single-cell cloning) to rule out the possibility that remaining epithelial cells within a mesenchymal population could take over due to their faster proliferation rate rather than an actual MET (Figure S1B). As a result, we were able to confirm that single mesenchymal cells were able to form colonies of epithelial cells after the withdrawal of TGFβ. Then, we examined the expression pattern of *Elf3* in NMuMG cells during the progression of EMT and MET. We have noticed that unlike the *Cdh1* expression pattern, *Elf3* RNA levels were relatively stable (Figure S1C). We then used several siRNAs targeting *Elf3* and validated their knockdown efficiency in NMuMG cells by measuring *Elf3* RNA levels. As a result, we noticed that all siRNAs used produced a similar phenotype with varying yet substantial knockdown efficiencies (Figure S2A). In cells transfected with nontargeting siRNA, we did not notice any morphological changes; on the other hand, *Elf3* targeting siRNAs caused a failure of MET evident by the enlarged and flattened cell morphology (Figure S2B). Confocal images of siElf3 transfected cells revealed oversized cells, with actin present as stress fibers, absence of E-cadherin at the plasma membrane, and an increased Vimentin expression (Figure 2). These results confirmed the MET failure and suggested that this aberrant MET was, in part, due to the absence of E-cadherin from the plasma membrane.

**Figure 3:**
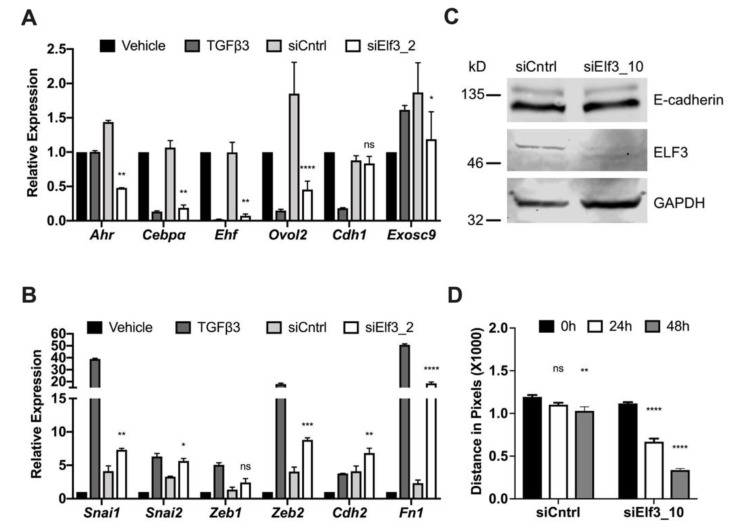
Loss of Elf3 preserves the mesenchymal state. NMuMG cells recovering from TGFβ3 treatment were transfected with either control or *Elf3* targeting siRNA, after 72 h, cells were collected, RNA was isolated, and changes in gene expression were measured by qPCR for EMT/MET relevant genes (**A**) and mesenchymal markers (**B**). (**C**) Immunoblot showing ELF3 and E-cadherin protein levels in response to *Elf3* silencing by siRNAs. (**D**) Summary of the wound healing assay results following *Elf3* silencing measured as the distance between the edges of migrating cells. Results represent the averages of at least three independent experiments in triplicates. Paired Student’s *t*-test was used to calculate statistical significance. * *p*-value < 0.05; ** *p*-value < 0.01; *** *p*-value < 0.001; **** *p*-value < 0.0001.

The enlarged cellular morphology was reminiscent of senescent cells, but we could not confirm this using the very well-established SA-β-Gal staining method (Figure S2C), suggesting a different cause for the enlarged cells. We wanted to confirm these observations by studying RNA expression levels of genes relevant to the EMT and MET programs. We selected *Ahr*, *Cebpα*, *Ovol2*, *Exosc9*, and *Ehf* for their potential involvement in the process of maintaining epithelial integrity, regulating the dynamic switch between epithelial and mesenchymal states, and key roles in metastasis [41,42,43,44,45]. NMuMG cells undergoing MET were transfected with different siRNAs targeting *Elf3*, and gene expression levels were evaluated by qPCR analysis. There was a significant downregulation of several transcription factors such as *Ahr*, *Cebpα*, *Ehf*, and *Ovol2* (Figure 3A).

On the other hand, the expression levels of some mesenchymal markers showed an evident increase in the absence of *Elf3*; we noticed a significant increase in the classical EMT inducers such as *Snai1/2* and *Zeb2* (Figure 3B), which was accompanied by an increased expression of *Cdh2* and *Fn1* (Figure 3B). In agreement with the data in Figure 2, RNA and protein levels of E-cadherin after *Elf3* depletion were comparable to those of control cells, which can complete the MET transition (Figure 3A,C). These results confirmed once more the MET failure, with a restricted appearance of the cells in a morphology reminiscent of sustained EMT. This mesenchymal state was also confirmed by the increased migratory capacity of *Elf3* depleted cells. We observed a five-fold increased migration as compared to control cells in wound healing assay (Figure 3D and Figure S3A). The observed impact on MET is not a typical feature of the depletion of transcription factors with a strong correlation with the epithelial phenotype. We affirmed this by using siRNAs targeting *Ehf*, another transcription factor from the Ets family, which is also found among the top 25 genes correlated with the epithelial phenotype. We found that the siRNA mediated depletion of *Ehf* did not affect MET progression nor the *Cdh1* RNA levels (Figure S3B,C, respectively).

### 3.3. Elf3 Regulates the Expression of Grhl3

**Figure 4:**
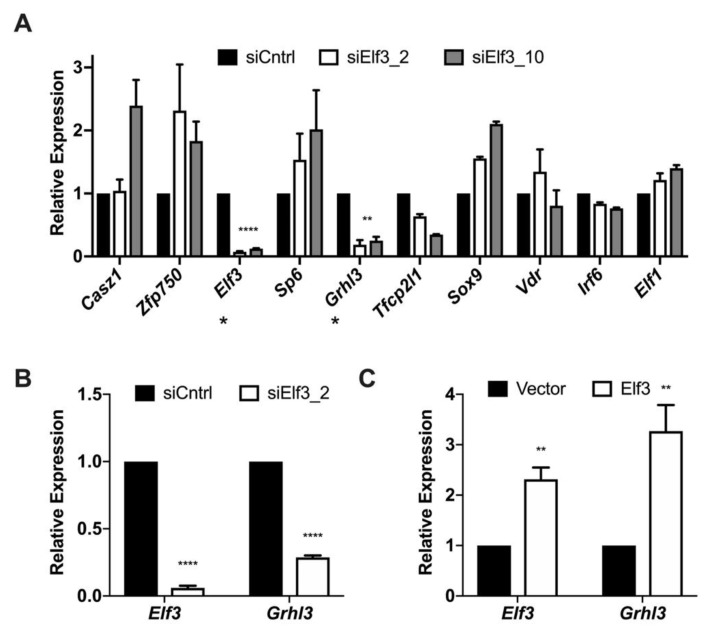
*Grhl3* expression levels change in response to modulating *Elf3* expression in different cellular models. (**A**) NMuMG cells recovering from TGFβ3 treatment were transfected with either control or *Elf3* targeting siRNAs, after 72 h, cells were collected, RNA was isolated, and changes in the expression of the top 10 genes correlated with the epithelial phenotype were measured by qPCR. The * below gene names indicate genes with efficient downregulation. (**B**) Primary mouse keratinocytes were transfected with siRNAs targeting *Elf3*. Seventy-two hours after transfection RNA was isolated, and the expression levels of *Elf3* and *Grhl3* were measured by qPCR. (**C**) MEFs were isolated from E13.5 embryos and transfected with ELF3 expressing plasmid. Seventy-two hours after transfection cells were collected in RNA lysis buffer, total RNA was isolated and converted to cDNA. qPCR was performed using *Grhl3* specific primers. Relative expression was calculated using the ΔΔCt method relative to the vector. Data represent the averages of at least three independent experiments. Paired Student’s *t*-test was used to calculate statistical significance. ** *p*-value = 0.005; **** *p*-value < 0.0001.

The results from Figure 3 revealed the effect of *Elf3* silencing on genes previously associated with epithelial integrity or EMT. We also wanted to study the influence of *Elf3* depletion on the genes highly correlated with the epithelial phenotype. For this, we examined the expression of the top 10 correlated genes from Figure 1 after *Elf3* silencing at the onset of MET. We noticed a significant downregulation of *Grhl3* expression and a modest downregulation of *Tfcp2l1* (Figure 4A). We were intrigued by the appearance of *Grhl3* in the top 5 genes correlated with *Cdh1* as well as its downregulation in response to *Elf3* silencing because of its importance in MET regulation [25]. For this reason, we measured z-scores for transcription factors correlated with *Grhl3*, and we observed that it also correlates with genes having the highest correlation with the epithelial phenotype, such as *Casz1* and *Elf3* (Figure S4A). We analyzed the bilateral correlation between *Elf3* and *Grhl3* in the datasets from Figure 1 and found a high correlation of 0.84, *p*-value < 2.22 × 10^−16^ (Figure S4B). We studied changes in *Grhl3* RNA levels after modulating *Elf3* expression in other cellular models and found that in primary keratinocytes, for example, *Grhl3* was significantly down-regulated upon *Elf3* depletion by siRNAs (Figure 4B). Also, the overexpression of ELF3 in primary MEFs was effective in increasing the RNA levels of *Grhl3* (Figure 4C), suggesting a potential transcriptional regulatory relationship between the two genes.

### 3.4. The Transcription Factor Elf3 Activates the Promoter of Grhl3

**Figure 5:**
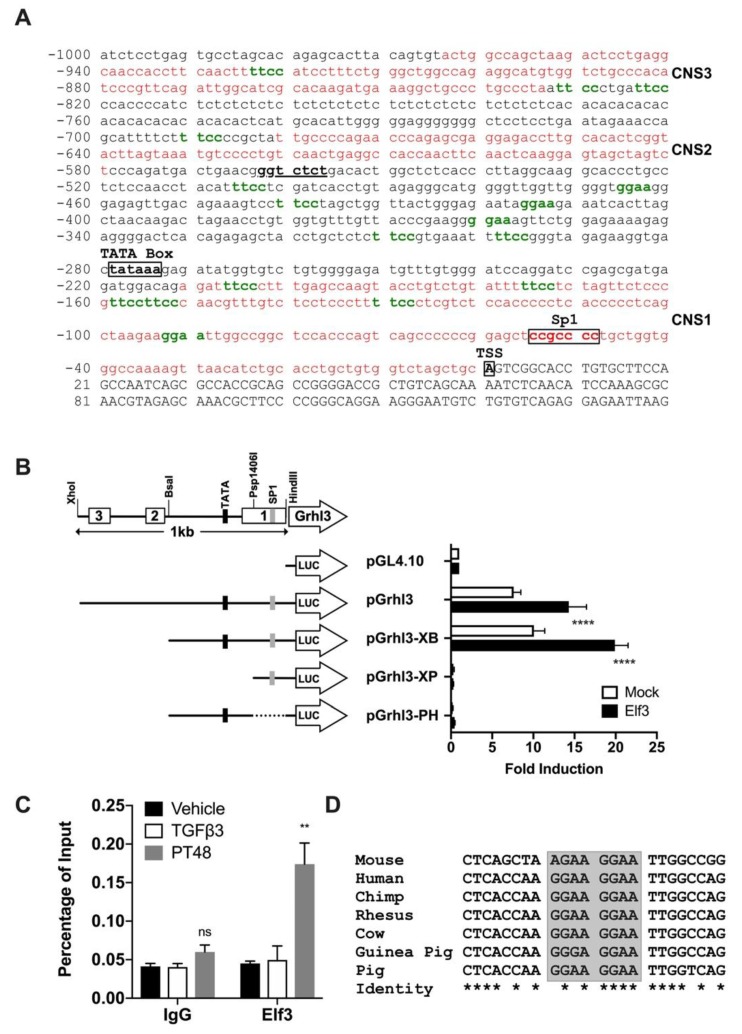
The transcription factor ELF3 regulates the *Grhl3* promoter. (**A**) The sequence of the *Grhl3* promoter (1000 bp) showing the location of the predicted TATA box, SP1 sequence, and the transcription start site. Sequences in red represent the conserved regions, and green bases represent putative ELF3 binding sites. (**B**) Luciferase reporter assay showing the activation of the *Grhl3* promoter by ELF3. Relative luciferase activity is calculated as fold induction relative to vector. Drawings on the left side show a schematic representation of the *Grhl3* promoter region (top) and the structure of each reporter plasmid (below). The conserved regions are shown as horizontal boxes labeled 1,2 and 3, the restriction enzymes used to generate the reporter deletions of the promoter are labeled. (**C**) ELF3 binding at the promoter of *Grhl3*. NMuMG cells were treated with either vehicle or TGFβ3 for 72 h, TGFβ3 treated cells were then washed with PBS and continued incubation for an additional 48 h to initiate MET (post-treatment: PT48). Cells were cross-linked, and chromatin immunoprecipitation was performed. ChIP DNA was used in qPCR to measure the occupancy of ELF3 compared to control antibodies. (**D**) Sequence alignment of one putative ELF3 binding site in the *Grhl3* promoter showing conservation among different species. In B and C, data represent the averages of at least three independent experiments. The paired Student’s *t*-test was used to calculate statistical significance. ** *p*-value < 0.01; **** *p*-value < 0.0001.

To address the potential transcriptional regulatory relationship between ELF3 and *Grhl3*, we analyzed the promoter region of *Grhl3* (1 kb proximal to TSS) for conserved sequences and ELF3 binding sites. The promoter of *Grhl3* was analyzed for conservation using the genome vista tools and aligned to several mammalian species. We were able to identify three conserved sequences from Mouse/Human and Mouse/Chimp alignments, and two conserved sequences from Mouse/Rhesus, Mouse/Cow, and Mouse/Dog alignments (Figure S5A). The sequence of the proximal promoter region is presented in Figure 5A, and some promoter elements are highlighted, the Sp1 site appeared conserved in all comparisons (Figure S5B). We identified a total of 17 ELF3 binding sites in the 1 kb region of *Grhl3* promoter (green nucleotides in Figure 5A). This 1 kb sequence was then cloned in the pGL4.10 vector to measure the reporter activity in response to ELF3 induction (Figure 5B). NMuMG cells were transfected with various extents of the promoter sequence; we observed that the *Grhl3* promoter retained its activity in the absence of CNS2 and 3 but required the presence of CNS1 together with the TATA and Sp1 elements (Figure 5B). Although the functionality of the TATA box per se cannot be deduced from these experiments, the presence of other crucial promoter elements cannot be ruled out

Moreover, we found that ELF3 expression was sufficient to upregulate the reporter activity. The activation of the *Grhl3* promoter by ELF3 was not confined to in vitro assays, as we were able to detect a significant enrichment of ELF3 at the *Grhl3* promoter in cells undergoing MET (Figure 5C). Of the 17 ELF3 binding sites in the *Grhl3* promoter, six were present in the first conserved sequence (CNS1: 210 bp upstream of TSS), four of which were found conserved among different mammalian species (Figure 5D and Figure S5D). The binding of ELF3 at the *Grhl3* promoter was confined to CNS1 during MET; we were not able to detect similar binding using primers outside of this conserved sequence (Figure S5C).

## 4. Discussion

The expression of the adhesion molecule E-cadherin is indispensable for the epithelial state, and its regulation is tightly and dynamically controlled during several developmental and physiological processes, during which EMT and MET programs alternate, and is characterized by the switching of cadherin gene expression. Positive regulation of *Cdh1* expression is a limiting factor for an efficient MET, suggesting that regulators of *Cdh1* are in fact regulators of MET and the epithelial state. Here, by using in silico methods and comparing several datasets with distinct epithelial and mesenchymal phenotypes, we defined an epithelial-specific set of transcription factors based on their correlation with *Cdh1* expression. We identified several transcription factors with high correlation with the epithelial phenotype, such as *Elf3*, *Grhl3*, and *Ovol2*. 

*Elf3*, a member of the Ets family of transcription factors, appeared to be essential for proper MET progression, and its depletion caused a severe MET defect marked by the absence of E-cadherin from the plasma membrane and also the downregulation of several epithelial genes in particular *Grhl3*. Closer examination of the relationship between *Elf3* and *Grhl3* led us to identify ELF3 as a regulator of *Grhl3* expression. ELF3 was able to activate the *Grhl3* promoter in reporter assays, and this was further verified as ELF3 was found to be present at the promoter of Grhl3 in cells undergoing MET.

The Ets family of transcription factors is one of the largest families of transcription factors, consisting of ~30 members (27 in humans and 26 in mice) that can be expressed both ubiquitously and in a tissue-specific manner [46,47]. Ets transcription factors are characterized by conserved winged helix–turn–helix DNA binding domains called ETS domain that binds a typical DNA sequence 5′-GGA(A/T)-3′ [46,48,49]. Members of this family are trans-acting phosphoproteins [50] that can act as upstream and downstream effectors of most signaling pathways, including MAP kinases, Erk1/2, p38, and JNK [51], thus playing crucial roles in a wide variety of biological processes such as differentiation, development, proliferation, apoptosis, tissue remodeling and the epithelial to mesenchymal transition with a suggested involvement in the mesenchymal to epithelial transition [52,53,54,55,56]. 

The E74-like transcription factor-3 *Elf3* (also known as ESX, ESE-1, ERT, and JEN), which was first described by Oettgen and colleagues in 1997 [57], is the main Ets transcription factor which is expressed ubiquitously by epithelial-rich tissues, such as stomach, pancreas, uterus, colon, kidney, lung, mammary gland, and skin [58,59]. The observed expression of *Elf3* in our EMT/MET model (Figure S1C) was not restricted to the epithelial states (Vehicle and PT) as for *Cdh1*, but also detected in the mesenchymal cells. While this is contradictory to the high correlation scores between *Elf3* and *Cdh1* in Figure 1, it could be explained by a context-dependent expression of *Elf3* in mesenchymal cells since previous reports indicated that it could also be induced in nonepithelial cells by the proinflammatory cytokines TNF-α and IL-1β [50,60]. This context-dependent expression of *Elf3* could also imply a context-dependent function of ELF3, the interaction of ELF3 with the *Grhl3* promoter was only detectable during MET, but not in mesenchymal cells.

Besides the critical roles of ELF3 in epithelial cell differentiation, gut development, inflammation, and apoptosis, it is also essential for the pathophysiology of cancer epithelial cells such as in breast, lung and prostate cancers [61,62,63,64]. It was demonstrated that ELF3 both activates the type II TGFβ receptor gene (*TβR-II*) in epithelial cells and binds *TβR-II* promoter in vivo [65]. For instance, ELF3 controls intestinal epithelial differentiation during development by altering the expression pattern of TβR-II [66]. ELF3 was also recently identified as a candidate transcriptional regulator involved in human urothelial cytodifferentiation together with GRHL3; its depletion in urothelial cells result in a declined expression of some transcription factors such as FOXA1 and GRHL3, which are known to be involved in urothelial differentiation [67]. It was reported that IRF6 and GRHL3 function downstream of RIPK4 to promote *ELF3* gene expression in keratinocytes [68], and considering the data presented here, it is plausible to hypothesize a positive feedback regulatory circuit involving *Grhl3* and *Elf3*. A recent publication also revealed that ZEB1 and ELF3 transcriptionally regulate *IRF6* [69].

The role of *Elf3* was also indicated in animal models. *Elf3* knockout results in embryonic lethality in ~30% of mice models at E11.5. Born *Elf3*^−/−^ mice develop a wasted phenotype characterized by watery diarrhea, lethargy, and malnourishment [66].

The studies mentioned above accentuated the importance of ELF3 in epithelial cells; this is in agreement with the findings presented here; *Elf3* tops the highly correlated transcription factors with the epithelial phenotype. The fact that *Grhl3* was also among the first factors correlated with the epithelial phenotype was not surprising; it holds a critical role in MET [25]. The close association between *Elf3* and *Grhl3* was reported in earlier studies [67], and hence, we deduced their regulatory relationship. The transcriptional relationship between *Elf3* and *Grhl3* was not limited to NMuMG cells since results obtained from MEFs and keratinocytes provided compelling proof. 

Similar to the loss of *Grhl3*, loss of *Elf3* during MET failed the epithelial transition. However, unlike *Grhl3*, lack of which resulted in downregulation of *Cdh1*, *Elf3* depletion did not cause a reduction in *Cdh1* mRNA or its protein levels. Instead, E-cadherin was absent from the plasma membrane, which is a significant obstacle preventing MET progression favoring a mesenchymal phenotype. The implications of a deregulated E-cadherin expression as well as its absence from the plasma membrane have been described before. Analysis of tumor samples suggested that aberrant expression of E-cadherin (or no expression) significantly correlates with poor prognosis and promoted metastasis [70,71,72]. However, the association of *Elf3* with an aberrant E-cadherin expression is a new finding but with comparable observations from Drosophila. The Drosophila aop (anterior open, also named Yan) and Eip74EF (Ecdysone-induced protein 74EF) are two genes found in the FlyBase [73] as orthologs for several mammalian Ets transcription factors, such as *Elf3*, *Ehf*, and *Elf5*. In particular, aop was reported to control the border cell migration in the Drosophila egg chamber by altering shg (DE-Cad) expression and its localization [74]. Therefore, a dramatic level of evolutionary conservation of the *Elf3* function cannot be ruled out. 

In Drosophila, grainy head (grh) is driving postembryonic neuroblast function and proliferation by promoting DE-cadherin expression [18,75]. This transcriptional relationship between grh and shg is conserved; the mouse orthologs of grh, GRHL2, and GRHL3 have been reported to regulate the transcription of *Cdh1* in mouse cells [25,31]. Interspecies functional conservation would then infer conserved regulation; this, in turn, would suggest conserved *cis*-regulatory elements, such as enhancers. Several reports have indicated that interspecies genome comparisons of non-coding DNA sequences could lead to correct predictions of regulatory sequences [25,40,76,77]. The presence of several conserved ELF3 binding sites in the promoter of *Grhl3* together with the experimental evidence on the regulatory effect of ELF3 on *Grhl3* is in support of the statement mentioned above.

Although the ETS transcription factors have a novel role in various diseases, the function of these transcription factors, particularly in cancer remains largely unclear. ELF3 depletion during MET was not limited to the aberrant expression of *Cdh1*; our data confirmed the role of ELF3 as a regulator of epithelial identity, reflected by the dramatic changes in the expression of selected EMT and MET relevant genes. The ligand-activated transcription factor Aryl hydrocarbon receptor (*Ahr*) promotes EMT and metastasis via its ligands l-kynurenine (l-Kyn) and d-kynurenine (d-Kyn) [45,78]. *EHF* has a regulatory function in epithelial cell differentiation and gaining stem cell-like properties via EMT [41]. The elevated expression of *EHF/ESE-3* is associated with poor clinical outcomes and increased recurrence [41,79].

Cellular plasticity is another big puzzle during EMT/MET phenotypic switches. Numerous studies have shown that the expression levels of EMT inducers are linked to cell plasticity and stem cell phenotypes. There is no doubt that the dynamic nature of EMT/MET transitions is highly regulated by other transcription factors which can influence known master EMT regulators. Multilevel regulation of some transcription factors defines a harmonic balance between epithelial plasticity and stability in a context-dependent manner. *OVOL2* was recently revealed as a transcriptional repressor of EMT through downregulating *ZEB1* and *TWIST* in various cancer types such as in lung, breast, and colorectal cancers [44,80,81]. In a recent report, OVOL2 was identified as a MET inducing transcription factor in fibroblasts, which also enhanced the reprogramming of dermal fibroblasts into keratinocyte-like cell state [82]. In this report *OVOL2* was identified following a comprehensive screening approach for transcription factors correlated with *CDH1* in a large number of expression datasets. In support of our findings, it was interesting to find that besides *Elf3* and *Ovol2*, similar transcription factors were also common between the two studies and present among the top correlated genes, such as *Grhl3*, *Irf6*, *Grhl2*, *Grhl1*, and *Ehf*, suggesting a conserved function of these transcription factors in EMT and MET regardless of the organism or cell type studied.

Considering the importance of EMT/MET during development and cancer, a better understanding of these fundamental processes will lead to a better comprehension of the common players in physiological and pathological contexts. In a recent study, *Elf5* emerged as a lineage regulator of mammary gland development and as an inhibitor of EMT in breast cancer via repressing Snail2/Slug [83]. Similarly, *Ovol2* was identified as an indispensable guardian of the epithelial differentiation besides its EMT suppressing ability [84]. Our findings present novel insights into the regulation of MET and seek to emphasize the remarkable role of *Elf3* as a transcriptional gatekeeper of epithelial state. It is also not surprising that physiological and pathological EMT share common attributes in terms of transcriptional regulators. Altogether, our findings provide a broader perspective for the management of metastatic cancers.

In conclusion, we provided compelling evidence for the involvement of ELF3 in the regulation of MET by altering the localization of E-cadherin. Moreover, ELF3 regulates the expression of *Grhl3* by binding to conserved sequences in its promoter. Loss of *Elf3* prevented the MET initiation and preserved the mesenchymal state. Further studies are needed to establish the mechanistic relevance of these findings as well as their relevance to possible functions of *Elf3* in the progression of metastasis.

## Supplementary Materials

The following are available online at https://www.mdpi.com/2073-4409/8/8/858/s1. Figure S1: The expression of Elf3 in NMuMG cells and during MET; Figure S2: The siRNA mediated silencing of Elf3 results in an impaired MET; Figure S3: Different influence of Elf3 and Ehf during the progression of MET; Figure S4: Genes correlated with Grhl3; Figure S5: Conservation of the Grhl3 promoter; Table S1: NCBI GEO accession numbers and phenotypes of samples used in correlation studies; Table S2: Sequence information of siRNAs used; Table S3: Primers and corresponding UPL probes used in qPCR experiments; Table S4: Sequences of oligonucleotides used for the cloning of the Grhl3 promoter; Table S5: qPCR primers and UPL probes used in ChIP experiments; Table S6: Primers used in RT-PCR experiments.
