# Supplementary material for: The Transcription Factor Elf3 Is Essential for a Successful Mesenchymal to Epithelial Transition
Source: Cells. 2019 Aug 9;8(8):858. doi: 10.3390/cells8080858 (PMC6721682; doi:10.3390/cells8080858)
Supplement: Supplementary file 1 [file cells-08-00858-s001.zip › Supplementary Figure Legends.pdf]

Figure S1. The expression of Elf3 in NMuMG cells and during MET. A) One micro grams of total RNA was isolated from NMuMG cells and was used to prepare cDNA to be used in RT-PCR experiments. The expression of *Casz1*, *Zfp750* and *Elf3* was visualized on agarose gels. *Tbp* was used as an internal control. In the upper gel, 2 µl of 1:20 diluted cDNA was used for PCR amplification, in the bottom gel, 2 µl of undiluted cDNA was used. **B**) Validation of MET after withdrawal of TGFβ3. NMuMG cells treated with TGFβ3 were washed and FACS sorted as epithelial or mesenchymal cells based on their E-cadherin expression. Single cells were allowed to grow in the absence of TGFβ to initiate MET (The upper panel). In the lower panel, cells treated with TGFβ for 72 hours were collected and seeded at a single cell density in fresh medium to initiate MET, formation of epithelial colonies was observed and documented at 24h intervals. **C**) The mRNA expression levels of *Elf3* are relatively stable when compared to those of *Cdh1* during the course of EMT/MET in NMuMG cells.

Figure S2. The siRNA mediated silencing of Elf3 results in an impaired MET. A) Efficiency of silencing was measured by qPCR analysis of RNA collected from NMuMG cells recovering from TGFβ3 treatment after *Elf3* knock-down using three different siRNAs. **B**) Phase contrast images of NMuMG cells showing the morphological changes that accompany the TGFβ3 mediated EMT-MET (upper panel), and the morphological changes associated with the silencing of *Elf3* (siElf3) as compared to control siRNA (siCntrl) at the onset of MET (lower panel). **C**) Phase contrast images of NMuMG cells and Huh7 cells showing that the phenotypic changes in *Elf3* depleted cells are not associated with senescence. NMuMG cells recovering from TGFβ3 were transfected with either control or *Elf3* targeting siRNAs, Huh7 cells were treated with Doxorubicin for 48 hours. All cells were subjected to SA-BGal staining. Scale bar is 100 µm.

Figure S3. Different influence of Elf3 and Ehf during the progression of MET. A) The absence of *Elf3* during MET preserves the mesenchymal state. NMuMG cells were transfected with either siCntrl or siElf3 and wound healing assay was performed, cells were monitored and documented at regular intervals. The red vertical lines define the original width of the scratch, and the green vertical lines show the edges of the migrating cells. **B**) **Healthy MET progression in the absence of *Ehf*.** NMuMG cells recovering from TGFβ3 treatment were transfected with siRNA targeting *Ehf*. Confocal images showing the typical localization of E-cadherin to the plasma membrane. Actin distribution (Phalloidin, green) and detection of E-cad (red). Nuclei are labeled with DAPI. Scale bar, 50 µm. **C**) mRNA levels of *Ehf* and *Cdh1* in cells transfected with siRNAs targeting *Ehf* show no changes in the expression of *Cdh1*.

Figure S4. Genes correlated with Grhl3. A) A plot showing the top 25 *Grhl3* correlated transcription factors in the epithelial and in the mesenchymal states. The x axis represents the differential expression of each transcription factor in epithelial versus mesenchymal samples. **B**) *Grhl3* and *Elf3* are highly correlated. The expression of *Grhl3* and *Elf3* was evaluated in each data set and the spearman correlation was calculated across all samples. *Grhl3* and *Elf3* showed a strong correlation and a strong co-regulation with the epithelial phenotype.

Figure S5. Conservation of the Grhl3 promoter. A) VISTA plot of sequence conservation in a 1 kb genomic DNA in human, mouse and rat. Percent nucleotide identities between mouse, human, chimp, rhesus, cow and dog DNA sequences are plotted as a function of position along the mouse sequence. Peaks of evolutionary conservation in overlapping exonic sequences are shaded blue. Aligned regions of >70% conservation over 80 bases are shaded pink. **B**) Sequence alignment of the Sp1 binding site in the *Grhl3* promoter showing conservation among different species. **C**) ELF3 is absent from the sequences outside of CNS1 of the *Grhl3* promoter. NMuMG cells were treated with either vehicle or TGFβ3 for 72 hours, TGFβ3 treated cells were then washed with PBS and continued incubation for an additional 48 hours to initiate MET (Post Treatment: PT48). Cells were cross-linked, and chromatin immunoprecipitation was performed. ChIP DNA was used in qPCR to measure the occupancy of ELF3 compared to control antibodies. **D**) Sequence alignment of putative ELF3 binding sites in the *Grhl3* promoter showing conservation among different species.
