# Supplementary material for: The Transcription Factor Elf3 Is Essential for a Successful Mesenchymal to Epithelial Transition
Source: Cells. 2019 Aug 9;8(8):858. doi: 10.3390/cells8080858 (PMC6721682; doi:10.3390/cells8080858)
Supplement: Supplementary file 1 [file cells-08-00858-s001.zip › Supplementary Tables.pdf]

Table S1. NCBI GEO accession numbers and phenotypes of samples used in correlation studies

Study GEO Accession	Phenotype	Sample
**GSE130548**	Epithelial	GSM3742048.CEL
**GSE130548**	Epithelial	GSM3742049.CEL
**GSE130548**	Epithelial	GSM3742050.CEL
**GSE130548**	Mesenchymal	GSM3742051.CEL
**GSE130548**	Mesenchymal	GSM3742052.CEL
**GSE130548**	Mesenchymal	GSM3742053.CEL
**GSE55072**	Epithelial	GSM1329077.CEL
**GSE55072**	Epithelial	GSM1329078.CEL
**GSE55072**	Mesenchymal	GSM1329081.CEL
**GSE55072**	Mesenchymal	GSM1329082.CEL
**GSE55711**	Epithelial	GSM1342162.CEL
**GSE55711**	Epithelial	GSM1342163.CEL
**GSE55711**	Mesenchymal	GSM1342164.CEL
**GSE55711**	Mesenchymal	GSM1342165.CEL
**GSE77551**	Epithelial	GSM2054383.CEL
**GSE77551**	Epithelial	GSM2054384.CEL
**GSE77551**	Epithelial	GSM2054385.CEL
**GSE77551**	Mesenchymal	GSM2054377.CEL
**GSE77551**	Mesenchymal	GSM2054378.CEL
**GSE77551**	Mesenchymal	GSM2054379.CEL

Table S2. Sequence information of siRNAs used.

siRNA	Sequences	Vendor
**siElf3_2**	TTGAACCAACTTGTTCGATAA	Qiagen
**siElf3_10**	GGUAUUACUACAAACGGGA	Dharmacon
**siElf3_12**	GGUUGGAGAGAGAGUCGGAAU	Dharmacon
**siCntrl**	Silencer™ Negative Control No. 1 siRNA	Dharmacon
**siEhf_1**	AACAATTTATGTTTAATGAAA	Qiagen
**siEhf_2**	ACCCTTGATCTATTTAATCAA	Qiagen
**siEhf_3**	AAGGAACACTACAGTTGATAA	Qiagen
**siEhf_4**	CAGCAAATGGATTCTGATCAA	Qiagen

Table S3. Primers and corresponding UPL probes used in qPCR experiments.

Primers	Forward	Reverse	Probe
**Ahr**	TGCACAAGGAGTGGACGA	AGGAAGCTGGTCTGGGGTAT	27
**Cdh1**	ATCCTCGCCCTGCTGATT	ACCACCGTTCTCCTCCGTA	18
**Cdh2**	TCCCTGAGATACAGCGTCACT	ATAATGAAGATGCCCGTTGG	17
**Cebpa**	AAACAACGCAACGTGGAGA	GCGGTCATTGTCACTGGTC	67
**Ehf**	TCATTGTCAAGACTGAACAAACC	GTCCAACAGATCTACTGTGCTACC	33
**Elf3**	ACCGAACCCTGACACACCT	AGCTGTACATGGCGTTGAAGT	46
**Exosc9**	CAAGGTGCCCCTATAGTGCT	GGTCTGAGCTCTTATTTTCTTTGG	106
**Fn1**	AGGTGGACCCCGCTAAAC	TGCCGCAACTACTGTGATTC	31
**Gapdh**	AGCTTGTCATCAACGGGAAG	TTTGATGTTAGTGGGGTCTCG	9
**Grhl3**	AAGGAAGATGTCGAATGAACTTG	TCGTCCTCATTACTGTAGGGAAA	100
**Ovol2**	GTGAGGATTGCGGCTACAC	TGGTCACTGTTCACATGCAG	79
**Snai1**	CTTGCTCCACAAGCACCA	GAGGATGGGGAGGTAGCAG	71
**Snai2**	ATCCTTGGGGCGTGTAAGT	TGAACCACTGTGATCCTTGG	6
**Zeb1**	TGGAGTTCAAAGGTTGTCGTT	TTGCCACATCAACACTGGTC	109
**Zeb2**	TTGCTCCAGGATGTGTGG	CACACACTTGTTTGTGTGCATATC	64
**Casz1**	CCACCTTTGACCCAGGAA	AGGCTCCTGCTTCACCTG	66
**Sox9**	GTACCCGCATCTGCACAAC	CTCCTCCACGAAGGGTCTCT	75
**Sp6**	TTCGGCCTAGGTCTCTTTCA	CCCACACACACACCTCATCT	32
**Tfcp2l1**	GGGGACTACTCGGAGCATCT	TTCCGATCAGCTCCCTTG	53
**Zfp750**	GAGCCAGCGTGAGAACAGA	CAGGAGAGTTCCTTCCGTCA	68
**Irf6**	GCTTGCTGCTCCTAACCTGA	CTTTCTGGTGGGCAATGAG	34
**Vdr**	CACCTGGCTGATCTTGTCAGT	CTGGTCATCAGAGGTGAGGTC	89
**Elf1**	CCAGAGGAAGCAACCATAGC	AACCTGGGTTGAAGCCTGTA	38

Table S4. Sequences of oligonucleotides used for the cloning of the Grhl3 promoter.

	**Forward**	**Reverse**
Grhl3 1kb Promoter	GCTAGCCTCGAGTGCCTAGCACAGAGCACTTAC	GGATCCAAGCTTGGTGCCGACTGCAGCTAGAC

Table S5. qPCR primers and UPL probes used in ChIP experiments

Primers	Forward	Reverse	Probe
**Grhl3_Prom_52**	TCTAGTTCTCCCGTTCCTTCC	TTCTTAGCTGAGGGGGTGAG	52
**Grhl3_Prom_108**	GGAGAAAAGAGAGGGGACTCA	TCACCTTCTCTACCCGGAAA	108

Table S6. Primers used in RT-PCR experiments

Primers	Forward	Reverse
**Casz1_RT**	TCTACTACCACGGCTGCCAC	TCGTTGCTGGATTCCTCGTG
**Zfp750_RT**	CGACTCCAGCAAGCTGAGCA	GGCGTTGCTTGCATACATGG
**Elf3_RT**	GTCTGGAGGGCAAGAAGAGC	CCAACCTCTTCTTCCTTCCA
**Tbp_RT**	AGCCTCAGTACAGCAATCAAC	GAACTTCACATCACAGCTCC
