# Supplementary figures and images for: The Transcription Factor Elf3 Is Essential for a Successful Mesenchymal to Epithelial Transition
Source: Cells. 2019 Aug 9;8(8):858. doi: 10.3390/cells8080858 (PMC6721682; doi:10.3390/cells8080858)

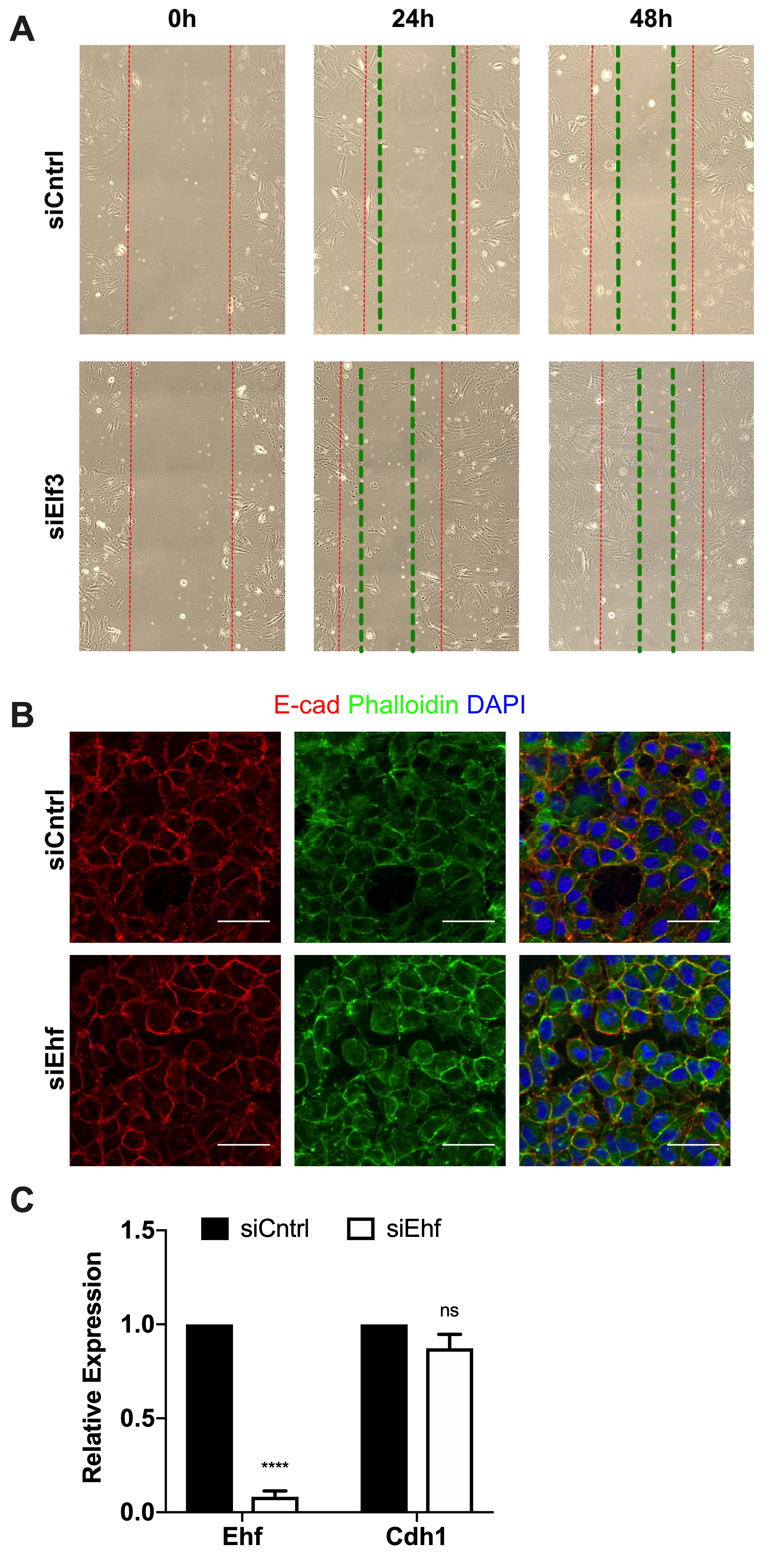

Supplement: Supplementary file 1 [file cells-08-00858-s001.zip › Figure S3.jpg]

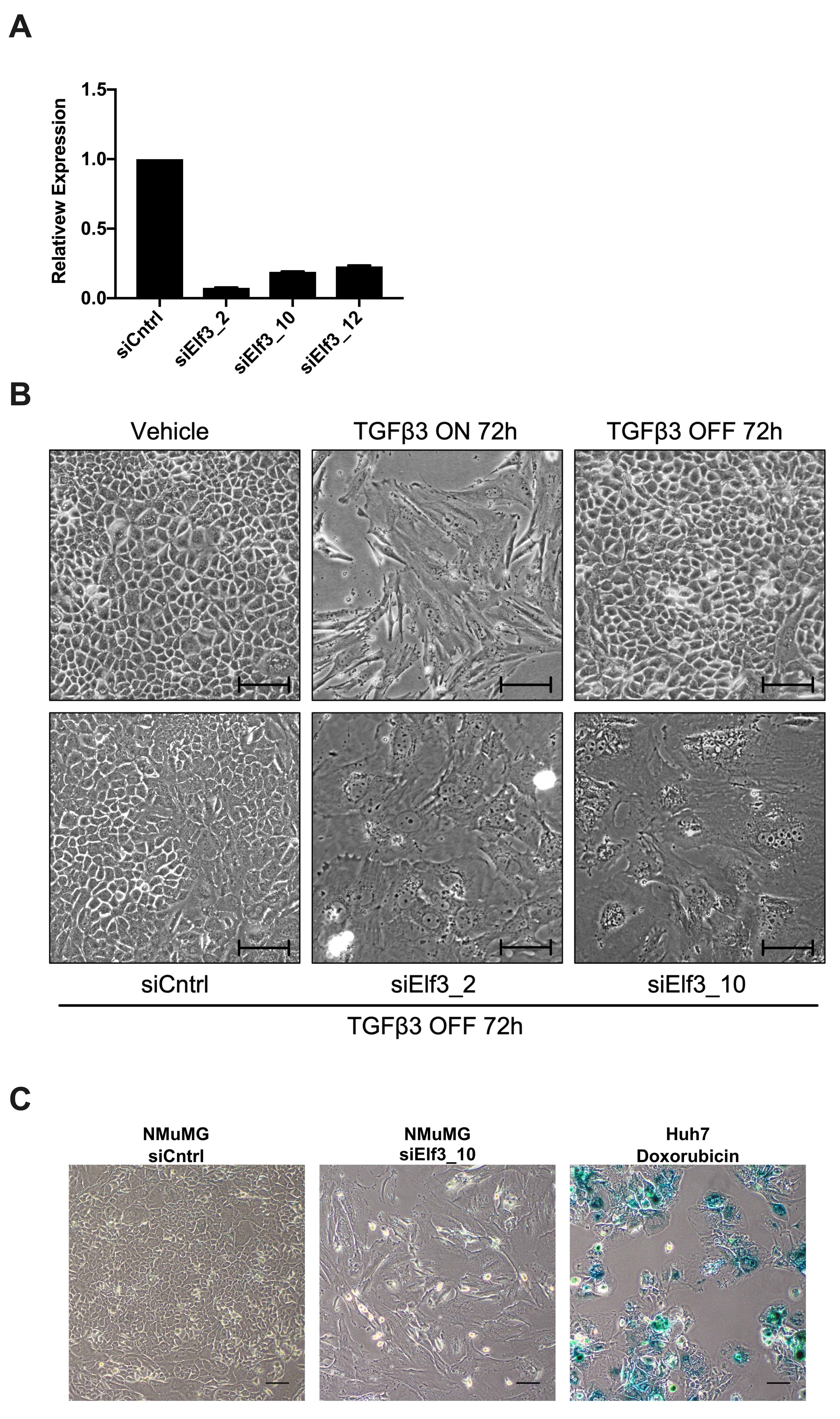

Supplement: Supplementary file 1 [file cells-08-00858-s001.zip › Figure S2.jpg]

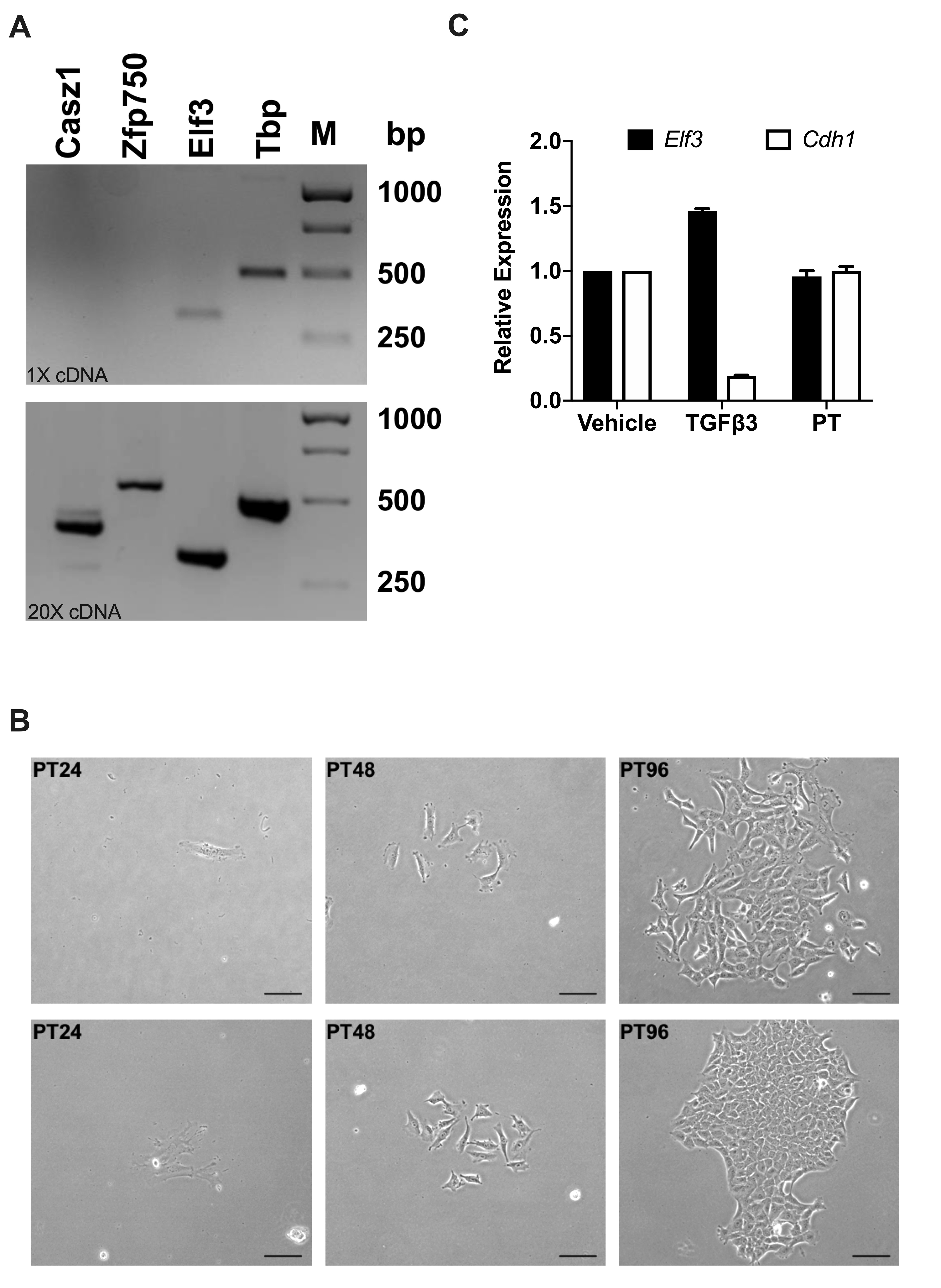

Supplement: Supplementary file 1 [file cells-08-00858-s001.zip › Figure S1.jpg]

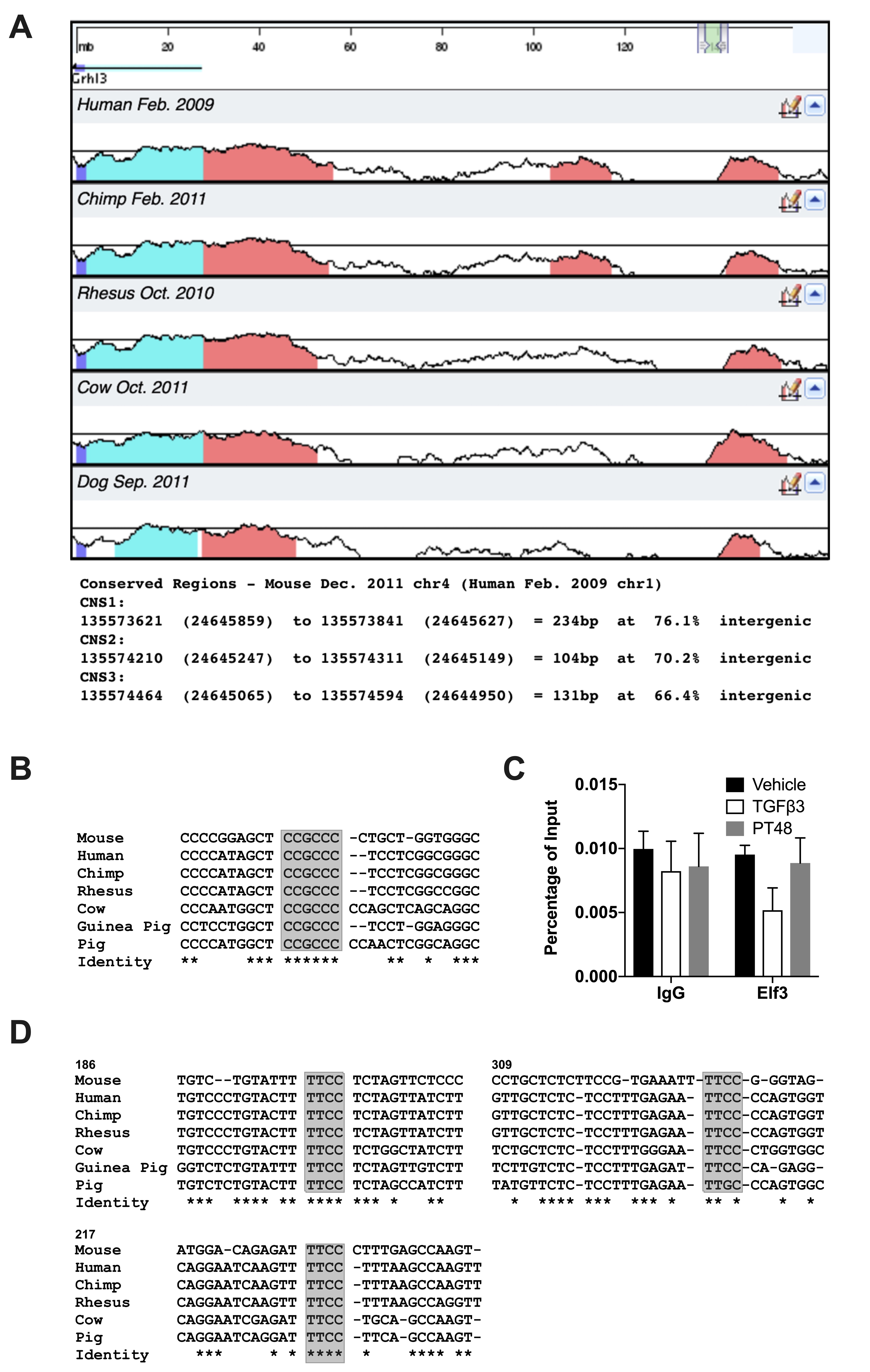

Supplement: Supplementary file 1 [file cells-08-00858-s001.zip › Figure S5.jpg]

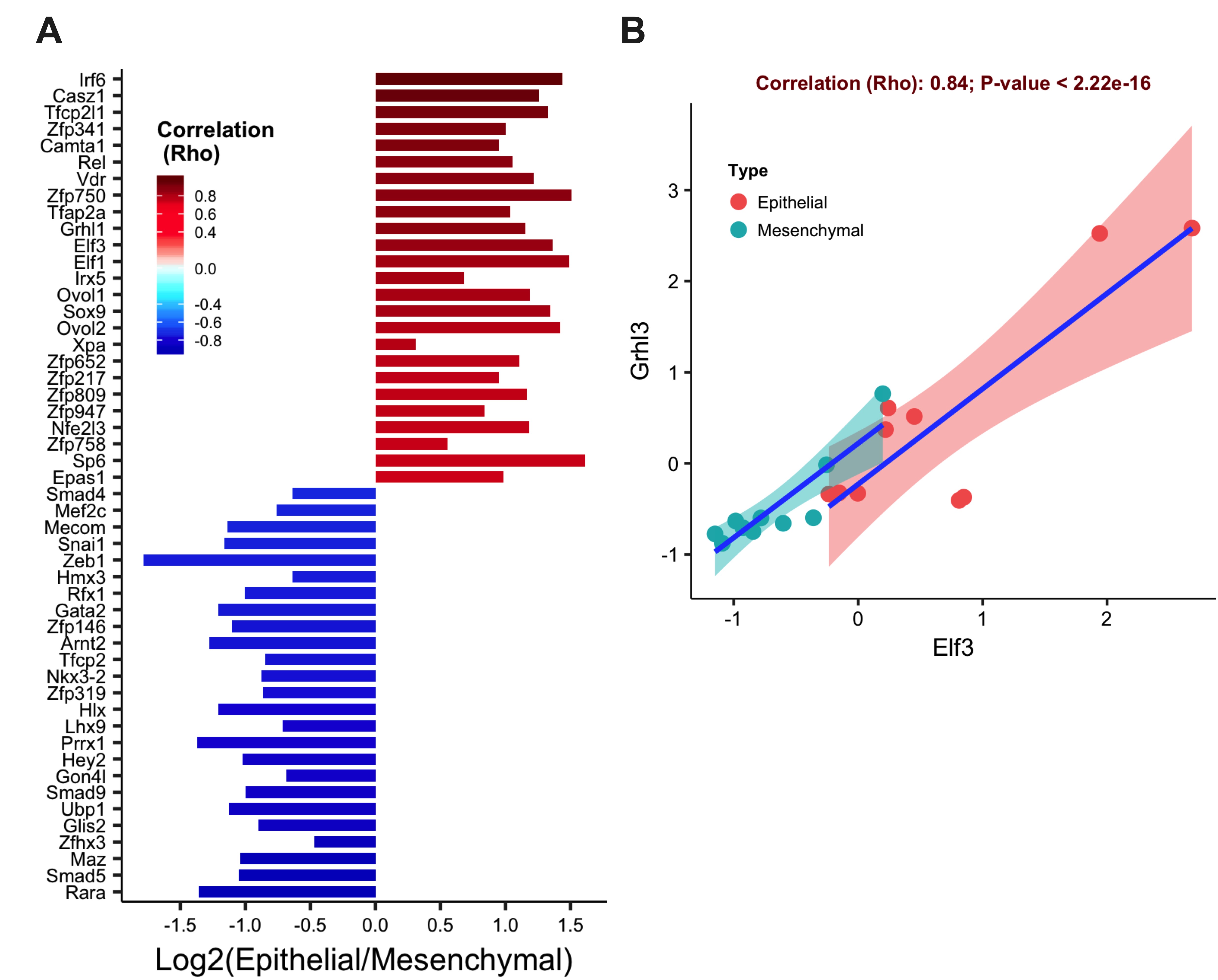

Supplement: Supplementary file 1 [file cells-08-00858-s001.zip › Figure S4.jpg]
